# LUBAC Formation Is Impaired in the Livers of Mice with MCD-Dependent Nonalcoholic Steatohepatitis

**DOI:** 10.1155/2015/125380

**Published:** 2015-06-10

**Authors:** Yasuka Matsunaga, Yusuke Nakatsu, Toshiaki Fukushima, Hirofumi Okubo, Misaki Iwashita, Hideyuki Sakoda, Midori Fujishiro, Takeshi Yamamotoya, Akifumi Kushiyama, Shin-ichiro Takahashi, Yoshihiro Tsuchiya, Hideaki Kamata, Fuminori Tokunaga, Kazuhiro Iwai, Tomoichiro Asano

**Affiliations:** ^1^Division of Molecular Medical Science, Department of Medical Chemistry, Graduate School of Biomedical Sciences, Hiroshima University, 1-2-3 Kasumi, Minami-ku, Hiroshima, Hiroshima Prefecture 734-8551, Japan; ^2^Division of Cervico-Gnathostomatology, Department of Dental Science for Health Promotion, Graduate School of Biomedical Sciences, Hiroshima University, Hiroshima, Hiroshima Prefecture 734-8551, Japan; ^3^Department of Internal Medicine, Graduate School of Medicine, University of Tokyo, 7-3-1 Hongo, Bunkyo-ku, Tokyo 113-0033, Japan; ^4^Division of Diabetes and Metabolism, The Institute for Adult Diseases, Asahi Life Foundation, 1-6-1 Marunouchi, Chiyoda-ku, Tokyo 100-0005, Japan; ^5^Department of Applied Biological Chemistry, Graduate School of Agricultural and Life Sciences, The University of Tokyo, Bunkyo-ku, Tokyo 113-0033, Japan; ^6^Laboratory of Molecular Cell Biology Institute for Molecular and Cellular Regulation, Gunma University, Maebashi, Gunma 371-8512, Japan; ^7^Department of Molecular and Cellular Physiology, Graduate School of Medicine, Kyoto University, Sakyo-ku, Kyoto 606-8501, Japan

## Abstract

Nonalcoholic steatohepatitis (NASH) is a disorder characterized by hepatic lipid accumulation followed by the inflammation-induced death of hepatocytes and fibrosis. In this process, oxidative stress contributes to the induction of several inflammatory cytokines including TNF-*α* andIL-1*β* in macrophages, while, in hepatocytes, NF-*κ*B reportedly induces the expressions of cell survival genes for protection from apoptosis. Recently, it was reported that the new ubiquitin ligase complex termed linear ubiquitin chain assembly complex (LUBAC), composed of SHARPIN (SHANK-associated RH domain-interacting protein), HOIL-1L (longer isoform of heme-oxidized iron-regulatory protein 2 ubiquitin ligase-1), and HOIP (HOIL-1L interacting protein), forms linear ubiquitin on NF-*κ*B essential modulator (NEMO) and thereby induces NF-*κ*B pathway activation. In this study, we demonstrated the formation of LUBAC to be impaired in the livers of NASH rodent models produced by methionine and choline deficient (MCD) diet feeding, first by either gel filtration or Blue Native-PAGE, with subsequent confirmation by western blotting. The reduction of LUBAC is likely to be attributable to markedly reduced expression of SHARPIN, one of its components. Thus, impaired LUBAC formation, which would result in insufficient NF-*κ*B activation, may be one of the molecular mechanisms underlying the enhanced apoptotic response of hepatocytes in MCD diet-induced NASH livers.

## 1. Introduction

Nonalcoholic steatohepatitis (NASH) is a serious liver disorder, which develops due to hepatic steatosis and then progresses to fibrosis, cirrhosis, and finally hepatocellular carcinoma [1]. At present, the “second hit theory” is the mostly widely accepted hypothesis for the molecular mechanism underlying NASH development. The first hit is fatty acid accumulation, and the second hit is the induction by inflammatory cytokines and oxidative stress, leading to hepatic fibrosis and cell death [2, 3].

In Kupffer cells, releases of several inflammatory cytokines including tumor necrosis factor-*α* (TNF-*α*), interleukin-6 (IL-6), and IL-1*β* are upregulated in NASH livers, and these expressions are reportedly regulated by nuclear factor-*κ*B (NF-*κ*B) transcriptional activity [4–6]. On the other hand, NF-*κ*B activity also reportedly induces the expressions of antiapoptosis factors such as inhibitors of apoptosis protein (IAPs), including X-linked IAP, which would promote the survival of hepatocytes [7, 8]. Thus, NF-*κ*B is involved in both inflammation and cell survival.

Linear ubiquitylation was recently shown to play an important role in the NF-*κ*B signaling pathway. Linear ubiquitin chains are formed on NF-*κ*B essential modulator (NEMO) by a linear ubiquitin chain assembly complex (LUBAC) and induce IKK activation [9–12]. LUBAC is comprised of HOIP, HOIL-1L, and SHARPIN [13–15]. Among these components, HOIP and HOIL have E3 ligase activity, but only HOIP serves as an E3 ligase when LUBAC is formed [16].

HOIP contains RING domain which binds with E2, NZF (ubiquitin binding zinc finger motifs of the Npl4 type) domain which binds with ubiquitin, and ubiquitin-associated (UBA) domain. The UBA domain of HOIP interacts with the ubiquitin like (UBL) domain of HOIL-1L [13–15]. In addition, interaction between HOIP and SHARPIN is mediated by the UBA domain of HOIP and the UBL domain of SHARPIN [13–15]. In SHARPIN-deficient cells, NF-*κ*B activation by TNF-*α* stimulation is reportedly impaired [17]. In addition, deficiencies of HOIP and HOIL attenuate NF-*κ*B signaling [11, 18]. Thus, HOIP, HOIL-1L, and SHARPIN are all necessary for efficient NF-*κ*B activation.

SHARPIN is expressed in many organs, including the liver, lungs, and colon, and SHARPIN-deficient mice exhibit chronic proliferative dermatitis [13–20], a phenotype which is attributed to a mutation which attenuates NF-*κ*B activity, leading to increased death of skin cells. Furthermore, NF-*κ*B signaling dysfunction in hepatocytes reportedly promotes the development of chronic inflammation, steatohepatitis, and hepatocellular cancer [21–23]. Similar to the changes affecting skin, more severe cell death was induced by TNF-*α* treatment in SHARPIN-deficient primary hepatocytes than in controls [17]. These results support a critical role for LUBAC in cell survival and in turn led us to consider the possibility that abnormalities of LUBAC formation in the liver are responsible for the death of hepatocytes in the NASH-affected liver.

Thus, in this study, we focused on the status of LUBAC formation in the methionine and choline deficient (MCD) diet-induced NASH liver and thereby obtained evidence of the impaired LUBAC formation in this NASH model.

## 2. Materials and Methods

### 2.1. Experimental Animals and Cell Culture

Male C57BL/6J (8 weeks) mice were fed a normal diet or the MCD diet for 8 weeks. The animals had unrestricted access to food and water, were housed in temperature and humidity controlled rooms, and were kept on a 12-hour light/dark cycle. The animal experiments were approved by the committee of Animal Experimentation, Hiroshima University. HepG2 cells were maintained at 37°C, in 5% CO_2_ in DMEM supplemented with 10% fetal calf serum and 100 U/mL penicillin and streptomycin.

### 2.2. Antibodies

Anti-SHARPIN, anti-HOIL-1L, and anti-HOIP antibodies were used as reported previously [10, 11, 14]. Anti-*α*-tubulin was purchased from Santa Cruz Biotechnology.

### 2.3. Histochemical Studies and TUNEL Staining

Paraffin-embedded sections were stained with hematoxylin and eosin (HE). TUNEL staining was performed, using the DeadEnd Fluorometric TUNEL System (Promega). Briefly, deparaffinized sections were treated with Pro K. After being washed, sections were reacted with terminal deoxynucleotidyl transferase and fluorescein-12-dUTP. After thorough washing, the slides were mounted using DAPI.

### 2.4. RT-PCR

Total RNA was extracted from the livers homogenized in TRIzol (Invitrogen) and reverse transcribed using the Verso cDNA synthesis Kit (Thermo). The protocol for the reverse transcription cycle was 42°C for 30 min and 95°C for 2 min. RT-PCR was performed using the CFX 96 Real-Time system (Bio-Rad) and SYBR Premix Ex Taq (TaKaRa). PCR was carried out in two steps, the first at 95°C for 5 seconds and the second at 60°C for 30 seconds, which were then repeated 40 times. Relative mRNA genes were normalized to the GAPDH mRNA level [24] and relative expression levels were determined by the comparative Ct method. The primers were as follows: GAPDH: tggaccaccaactggttagc and ggcatggactgtggtcatgag, TNF-*α*: gaactggcagaagaggcact and agggtctgggccatagaact, IL-1*β*: aggagaaccaagcaacgaca and tgcttgtgaggtgctgatgt, Xiap: ccgggaggagctatctatca and tgaccagatgtccacaagga, and IAP: tgacgtgtgtgacaccaatg and tgaggttgctgcagtgtttc.

### 2.5. Immunoblotting

Livers were homogenized in lysis buffer (50 mM Tris, 1 mM EDTA, 1 mM EGTA, 150 mM NaCl, 50 mM NaF, 1% Triton X-100, 1 mM DTT, and 1 mM PMSF). After centrifugation, cell lysates were subjected to SDS-PAGE and transferred to a PVDF membrane. After blocking in phosphate buffered saline (PBS) containing 0.1% Tween 20 and 3% skim milk for 1 hr, the membrane was incubated with the appropriate primary antibody (1 : 1000) for 1 hr, followed by incubation with ECL anti-rabbit/mouse IgG Horseradish Peroxidase linked whole antibody (1 : 2000) for 1 hr. After washing with PBS-T three times, bands were detected using Super Signal West Pico Stable Peroxidase Solution (Thermo) on X-ray films.

### 2.6. Gel Filtration

Liver sections were lysed in lysis buffer (50 mM Tris-HCl, pH 7.5, 1 mM MgCl_2_, 1 mM DTT, and 1 mM PMSF, protease inhibitor cocktail). Since the LUBAC is located in the cytosolic fraction, as reported previously, detergent is not required [14]. After adding an equal volume of detergent-free lysis buffer containing 300 mM NaCl, lysates were centrifuged at 100,000 g for 30 min. The supernatants were then applied to gel filtration using a Superdex 200 10/300 GL (GE Healthcare) and fractionated at 1 mL/min in elution buffer containing 50 mM Tris-HCl pH 7.5 and 150 mM NaCl using an AKTA explorer 10S (GE Healthcare). The collected samples were subjected to immunoblotting analysis.

### 2.7. Blue Native PAGE

Blue native PAGE was performed according to the instructions for the NativePAGE Novex Bis-Tris Gel System (Invitrogen), in which addition of detergent (digitonin) is required to avoid protein aggregation. Livers were homogenized in 4× NativePAGE Sample Buffer (50 *μ*M Bis Tris, 0.1% Glycerol, 50 *μ*M NaCl, and 0.00001% Ponceau S) and 1% digitonin. After the lysates had been centrifuged at 15,000 rpm, 5% G-250 was added to the supernatants which were then electrophoresed using NativePAGE Novex3-12% Bis-Tris Gel (Invitrogen). The gels were then incubated in 0.1% SDS and transferred to a PVDF membrane (Millipore). After blocking in PBS containing 0.1% Tween 20 and 3% skim milk, the membrane was incubated with the appropriate primary antibody, followed by incubation with ECL anti-rabbit/mouse IgG Horseradish Peroxidase linked whole antibody (GE Healthcare).

### 2.8. Statistical Analysis

Data are expressed as means ± SEM. Student's *t*-test was employed for all analyses and values of *p* < 0.05 were considered to indicate a statistically significant difference.

## 3. Results

### 3.1. Preparation of MCD Diet-Induced NASH Rodent Model

**Figure 1:**
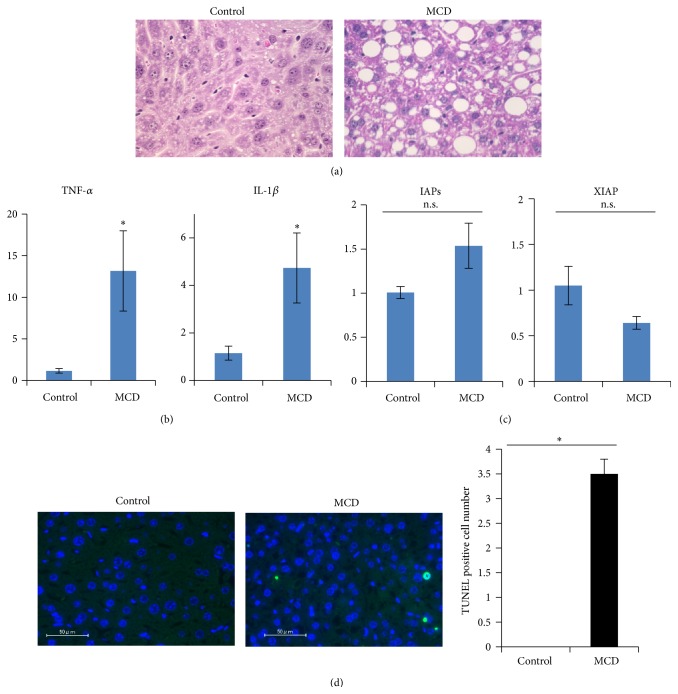
Eight-week-old mice were fed the control or the MCD diet for 8 weeks. Their livers were removed and then used for the experiments. (a) Paraffin-embedded sections were stained with hematoxylin and eosin. (b, c) RNA was isolated from the livers and mRNA levels of inflammatory cytokines (TNF-*α* and IL-1*β*) and antiapoptosis factors (IAPs and XIAP), regulated by the NF-*κ*B transcriptional activity, were determined by real-time PCR using primers corresponding to their mRNA sequences (*n* = 5). The data were normalized with the expression of GAPDH mRNA. (d) Apoptotic cell death in livers was detected by TUNEL staining. The data shown are representative of three to five independent experiments and shown as means ± S.E.M. ^*∗*^ *p* < 0.05.

C57BL/6J mice were fed the MCD diet for 8 weeks. Their livers were harvested and subjected to histological analysis. HE staining revealed marked increases in fat droplets and increased inflammatory cell infiltration and balloon-like structures in the livers of MCD diet-fed mice (Figure 1(a)). Expressions of inflammation cytokines such as TNF-*α* and IL-1*β* in the livers were markedly increased, reflecting the presence of inflammation (Figure 1(b)). Next, we examined the hepatic expression levels of IAPs and XIAP, which are regulated by NF-*κ*B transcriptional activity and are also known to be antiapoptosis factors, using RT-PCR. As shown in Figure 1(c), the expression levels of IAPs and XIAP were revealed to be similar to those of the controls. In addition, MCD diet feeding increased the number of apoptotic cell deaths detected by TUNEL staining (Figure 1(d)).

### 3.2. LUBAC Formation Was Severely Impaired in MCD Diet-Induced NASH Livers

**Figure 2:**
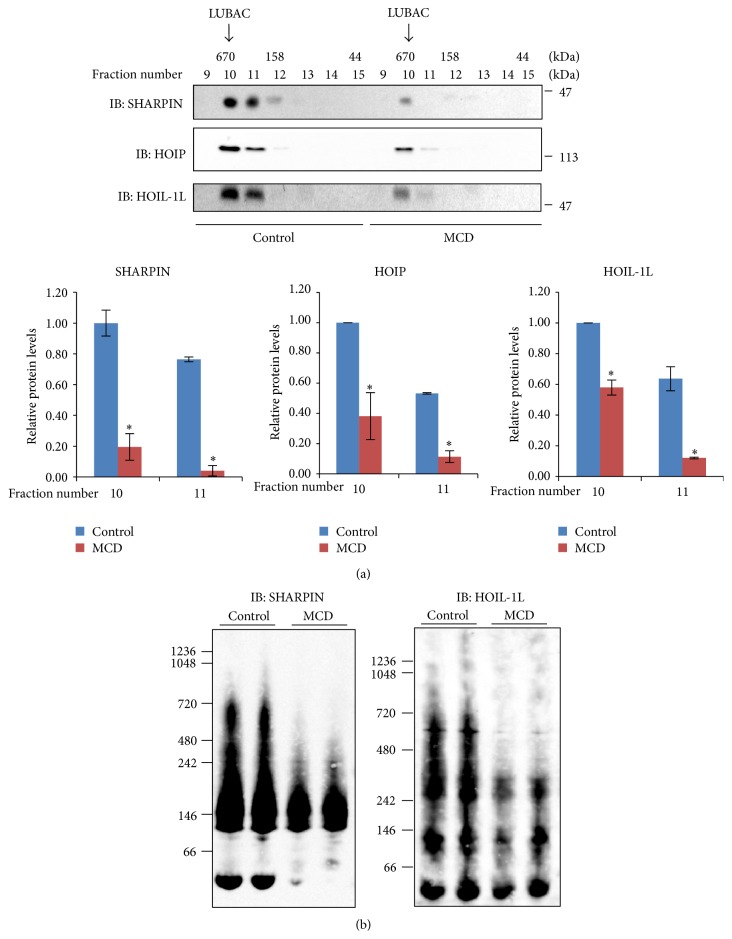
Eight-week-old mice were fed the control or the MCD diet for 8 weeks. Their livers were removed and LUBAC formation was investigated. (a) The liver cell lysates were subjected to gel filtration. Each fraction was analyzed by western blotting with anti-HOIP, anti-HOIL-1L, or anti-SHARPIN antibody. (b) The liver cell lysates were subjected to Blue Native-PAGE without SDS, followed by western blotting with anti-HOIL-1L or anti-SHARPIN antibody. The immunoblotting data are representative of three to five independent experiments.

As mentioned in Section 1, LUBAC consisting of HOIP, HOIL-1L, and SHARPIN plays a critical role in NF-*κ*B transcriptional activation. We investigated LUBAC formation in NASH livers employing gel filtration followed by immunoblotting as well as Native-PAGE electrophoresis. First, livers were homogenized in lysis buffer without detergent and centrifuged at 100,000 g. After gel filtration, the fractionated samples were subjected to immunoblotting with anti-HOIP, anti-HOIL-1, or anti-SHARPIN antibody (Figure 2(a)). HOIP and HOIL-1 detected in the fraction with sizes of approximately 600 kDa were markedly reduced in the NASH livers, as compared with the controls (upper and middle panels of Figure 2(a)). SHARPIN was present in the fractions of approximately 600 kDa and lower molecular weights, and its expression was also markedly lower in the livers of mice fed the MCD diet than in those of mice given the control diet (lower panel of Figure 2(a)).

Considering that LUBAC should contain all three of the aforementioned components, that is, HOIP, HOIL-1L, and SHARPIN, the band of approximately 600 kDa was considered to correspond to LUBAC. Comparison between the samples of MCD diet-fed and control mice revealed this band corresponding to LUBAC to be significantly reduced in the former, regardless of whether anti-HOIP, anti-HOIL-1L, or anti-SHARPIN was used, although the anti-SHARPIN immunoblotting data provided the most convincing evidence of this reduction (Figure 2(a)).

In order to further confirm the reduced LUBAC formation, we employed another method. Blue Native-PAGE was used without detergent or a heating procedure, and this was then followed by immunoblotting. With Blue Native-PAGE, the bands are not as sharp as those with routine SDS-treated electrophoresis. While anti-HOIP antibody failed to recognize HOIP after Blue Native-PAGE, immunoblotting using anti-HOIL-1L and anti-SHARPIN antibodies clearly showed the reduction in the approximately 600 kDa band corresponding to LUBAC in the lysates of MCD diet-fed mouse livers (Figure 2(b)). These results indicate LUBAC formation to be severely impaired in the livers of mice fed the MCD diet.

### 3.3. SHARPIN Expression Is Reduced in the Livers of Mice Fed the MCD Diet

**Figure 3:**
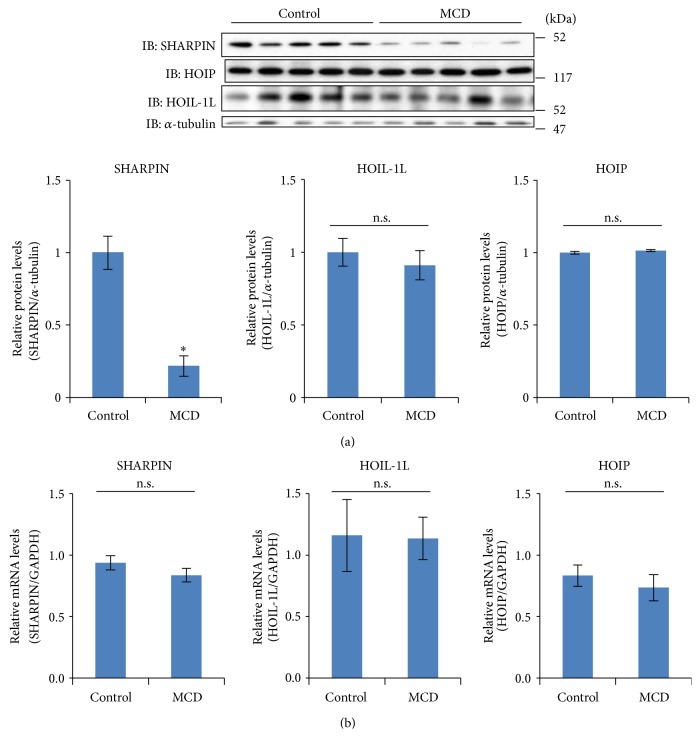
(a) Eight-week-old mice were fed the control or the MCD diet for 8 weeks (*n* = 5 for each group). Their livers were homogenized in lysis buffer and cell lysates were separated by SDS-PAGE. Expressions of HOIP, HOIL-1L, and SHARPIN were determined by western blot using the corresponding antibodies. Each lane in the immunoblot corresponds to a different mouse. The quantitative SHARPIN/*α*-tubulin data are shown in the bar graph. Data are shown as means ± S.E.M. ^*∗*^ *p* < 0.05. (b) RNA was isolated from the livers and mRNA levels of SHARPIN, HOIL-1L, and HOIP were determined by real-time PCR using primers corresponding to their mRNA sequences (*n* = 5). The data were normalized with the expression of GAPDH mRNA.

Since LUBAC formation was markedly reduced in the livers of mice with MCD diet-induced NASH, we examined the expression levels of SHARPIN, HOIL-1L, and HOIP separately (Figure 3). The immunoblotting with each antibody revealed SHARPIN expression to be markedly reduced, by approximately 80%, while those of HOIL-1L and HOIP were not significantly altered. The SHARPIN mRNA level did not differ between the NASH and control livers (Figure 3(b)).

### 3.4. Treatment with Palmitate Reduced SHARPIN

**Figure 4:**
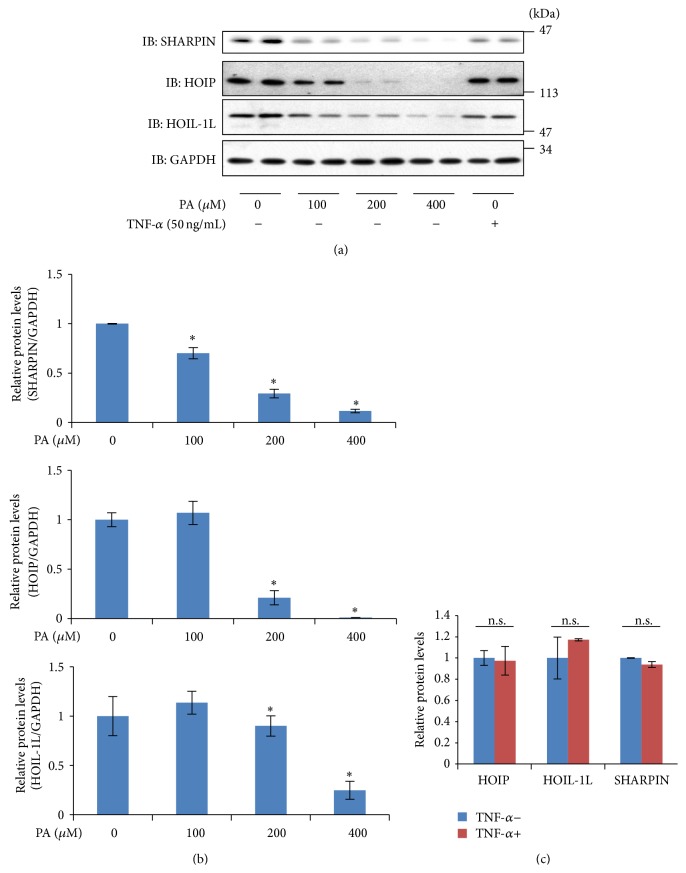
HepG2 cells were incubated for 24 hr in the presence or absence of PA or TNF-*α*. (a) Cell lysates were harvested in lysis buffer and expressions of HOIP, HOIL-1L, and SHARPIN were determined by immunoblotting using the corresponding antibodies. (b) The expression changes in LUBAC components with palmitate stimulation are shown in the bar graph. (c) The expression changes in LUBAC components with TNF stimulation are presented as quantitative data. These data are representative of three to five independent experiments, and results are shown as means ± S.E.M. ^*∗*^ *p* < 0.05.

To investigate the molecular mechanism underlying the reduced SHARPIN expression, we examined the effects of palmitate and TNF-*α* (50 ng/mL) on the expression of SHARPIN using HepG2 cells, since hepatocytes would be exposed in vivo to excessive fatty acids and inflammatory cytokines. After stimulation with palmitate or TNF-*α* for 24 h, the expression levels of SHARPIN, HOIL-1L, and HOIP were examined by immunoblotting (Figure 4). Stimulations with 100 *μ*M and 200 *μ*M of palmitate reduced SHARPIN by approximately 50% and 70%, respectively. In contrast, HOIL-1L expression levels were unaltered by either 100 *μ*M or 200 *μ*M of palmitate. HOIP was unaltered by 100 *μ*M palmitate but reduced by 200 *μ*M palmitate. In addition, 50 nM of TNF-*α* did not affect the expression levels of SHARPIN, HOIL-1L, or HOIP. These observations suggest that the lipotoxicity associated with palmitate accumulation may reduce SHARPIN expression in the liver.

### 3.5. Treatment with Palmitate Induces Proteasomal Degradation of SHARPIN

**Figure 5:**
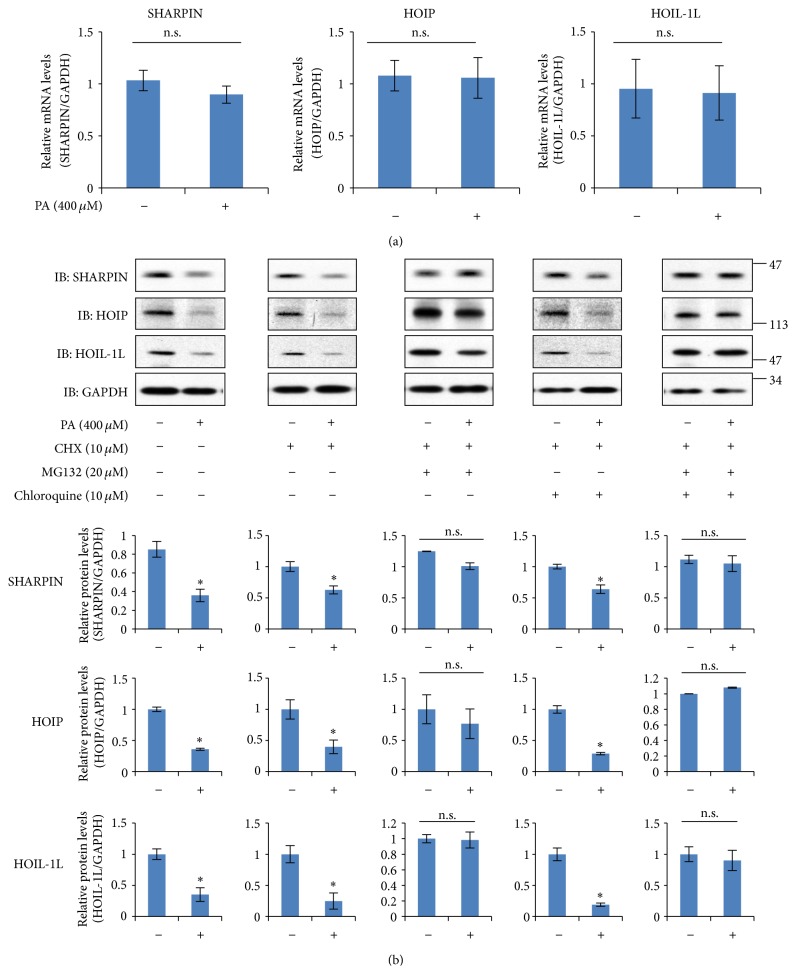
(a) HepG2 cells were incubated in the presence or absence of palmitate. After incubation for 12 hr, total RNA was extracted and reverse transcribed, and the mRNA levels of SHARPIN, HOIP, and HOIL-1L were quantified by real-time PCR. As the housekeeping gene, GAPDH was used for quantity normalization. The results shown are means ± S.E.M. (*n* = 5). n.s.: not significant. (b) HepG2 cells were incubated with the indicated combination of 20 *μ*M MG132, 10 *μ*M chloroquine, and 10 *μ*M cycloheximide, in the presence or absence of palmitate for 24 hr. Cell lysates were harvested in lysis buffer and expressions of SHARPIN, HOIP, and HOI-1L were determined by immunoblotting using the corresponding antibodies. For each combination, a representative immunoblot is shown and quantified data are expressed as the percentage ± S.E.M of five independent experiments. (*n* = 5). n.s.: not significant.

To elucidate the mechanism underlying the palmitate-induced downregulation of SHARPIN, we first assessed the expression levels of SHARPIN, HOIP, and HOIL-IL mRNA in HepG2 cells exposed to palmitate (Figure 5(a)). Real-time PCR analysis revealed that the mRNA levels of SHARPIN, HOIP, and HOIL-1L were not affected by treatment with 400 *μ*M palmitate for 12 h.

Next, we performed experiments to examine degradation in response to palmitate stimulation. After treatment of HepG2 cells with the translation inhibitor cycloheximide, the relative SHARPIN, HOIP, and HOIL-1L levels were determined by immunoblotting (Figure 5(b)). The levels of SHARPIN, HOIP, and HOIL-1L decreased more markedly in the cells treated with 10 *μ*M cycloheximide and 400 *μ*M palmitate than in those exposed to 10 *μ*M cycloheximide alone, which suggests the involvement of degradation rather than synthesis in the observed palmitate-induced downregulation. To distinguish the contributions of proteasome and lysosome pathways, HepG2 cells treated with the proteasome inhibitor MG-132 (20 *μ*M), chloroquine (10 *μ*M), or both were stimulated with 400 *μ*M palmitate (Figure 5(b)). Interestingly, palmitate-inducible downregulations of SHARPIN, HOIL-1L, and HOIP were significantly attenuated by coincubation with MG132, but not by that with chloroquine. This result suggests the palmitate-induced downregulations of SHARPIN, HOIL-1L, and HOIP to be mediated via proteasomal degradation.

## 4. Discussion

LUBAC consists of HOIP, HOIL-1L, and SHARPIN and produces linear ubiquitin on NEMO associated with IKK*α*/*β* [13–15]. Inflammatory cytokines such as TNF-*α* induce IKK activation and activated IKK phosphorylates IkB*α*. Then, while IkB*α* is degraded by proteasomes, NF-*κ*B translocates into the nucleus [25]. NF-*κ*B activation in hepatocytes reportedly functions to protect the liver from injury, steatosis, and hepatocellular carcinoma development. For example, mice lacking NEMO in hepatocytes develop steatohepatitis and carcinoma [23], as well as exhibiting severe liver damage when injected with TNF [22]. Interestingly, hepatocyte specific IKK2/*β* deletion does not increase sensitivity to either TNF or lipopolysaccharide [21]. Taking these reports into consideration, we speculate that dysregulation of NEMO sensitizes hepatocytes to cytokines or reactive oxygen species in the NASH state. We thus performed this study focusing on LUBAC, a newly recognized complex that regulates NEMO.

In this study, impaired LUBAC formation was demonstrated in the livers of MCD diet-fed mice, using two methods, that is, gel filtration and Blue Native-PAGE. LUBAC deficiency was considered to be attributable mainly to markedly reduced expression of SHARPIN. In addition, the experiments using HepG2 cells suggest that free fatty acids such as PA may be involved in the reduced level of SHARPIN protein expression. Since the palmitate-induced downregulation of SHARPIN was prevented under conditions of coincubation with MG132, this reduction in the protein level is very likely to be attributable to alterations in proteasomal degradation processes. Indeed, Ishii et al. suggested ubiquitination and proteasomal degradation to be enhanced by exposing HepG2 to palmitate [26]. We speculated that palmitate enhances ubiquitination of these molecules and thereby evokes both functional defects and, ultimately, their degradation.

Regarding the molecular mechanisms underlying the development of NASH, the two-hit theory has now been widely accepted [2, 3]. While the first hit is lipid accumulation, the second hit is inflammation and fibrosis induced by the releases of inflammatory cytokines and oxidative stress [1]. In hepatocytes, stimulation with TNF-*α* triggers activations of both JNK-mediated and IKK-mediated pathways. While the JNK-mediated pathway activation promotes cell death, NF-*κ*B pathway activation reportedly contributes to cell survival [27, 28]. These NF-*κ*B-induced antiapoptotic factors may function to protect hepatocytes against inflammation and oxidative stress in the NASH liver. In fact, Sieber et al. demonstrated TNF-*α* induced activation of NF-*κ*B to be strongly reduced in hepatocytes from SHARPIN-deficient mice, and injection of lipopolysaccharides induced more severe liver damage in SHARPIN-deficient mice than in controls [17]. Thus, they concluded that the survival pathway activation mediated by TNF-*α* is decreased in SHARPIN-deficient mice, while cell death pathway activation is increased such that the livers of these animals show necrosis [17].

Similar to the observations in SHARPIN-deficient mice, HOIL-1-deficient mice also showed increased apoptosis of hepatocytes with expressions of NF-*κ*B targeting genes being reduced, when treated with TNF-*α*, with enhanced JNK activity [11]. The portal and perivenous areas in the livers of SHARPIN-deficient mice were infiltrated by neutrophils and macrophages, and TNF-*α* ablation reversed inflammatory liver phenotypes such as granulocyte accumulations in SHARPIN-deficient mice [15]. Thus, it is very likely that LUBAC functions to suppress the inflammation and cell death triggered by TNF-*α* stimulation. We can tentatively speculate that hepatic fatty acid accumulation, as a first hit, contributes to decreased expression of SHARPIN and thereby failure of LUBAC assembly. This leads to impaired NF-*κ*B activation in hepatocytes. Therefore, hepatocytes in the fatty liver may be vulnerable to oxidative stress and/or inflammatory cytokines as the second hit.

In conclusion, this is the first study demonstrating impaired LUBAC formation to possibly be due to reduced expression of SHARPIN in the NASH rodent model liver, and this impairment in LUBAC formation may be one of the causes of hepatocyte death via suppressed expression of antiapoptotic factors regulated by NF–*κ*B activation.

## Abbreviations

LUBAC: Linear ubiquitin chain assembly complex
SHARPIN: SHANK-associated RH domain-interacting protein
HOIL-1L: Longer isoform of heme-oxidized iron-regulatory protein 2 ubiquitin ligase-1
HOIP: HOIL-1L interacting protein.

## Highlights

1. Linear ubiquitin chain assembly complex (LUBAC) plays a critical role in NF-*κ*B activation.
2. Formation of LUBAC is markedly impaired in the NASH rodent model liver, mainly because of the reduced SHARPIN expression.
3. Incubation with palmitate reduced the amounts of SHARPIN, HOIP, and HOIL-1L, possibly via proteasomal degradation in HepG2 cells.
